# Anti-Invasive and Anti-Proliferative Effects of shRNA-Loaded Poly(Lactide-Co-Glycolide) Nanoparticles Following ***RAN*** Silencing in MDA-MB231 Breast Cancer Cells

**DOI:** 10.1007/s11095-018-2555-6

**Published:** 2018-12-17

**Authors:** Ankur Sharma, Paul McCarron, Kyle Matchett, Susan Hawthorne, Mohamed El-Tanani

**Affiliations:** 1 School of Pharmacy and Pharmaceutical Sciences, Ulster University, Cromore Road, Coleraine, Co. Londonderry BT52 1SA UK; 2 School of Medicine, Dentistry and Biomedical Sciences, Queen’s University Belfast, Health Sciences Building, 97 Lisburn Road, Belfast, BT9 7BL UK; 3 Institute of Cancer Therapeutics, ICT building, University of Bradford, Richmond Road, Bradford, England BD7 1DP UK

**Keywords:** Breast cancer, Intracellular delivery, Nanotechnology, PLGA, shRNA

## Abstract

**Background:**

Overexpression of the RAN GTP (*RAN*) gene has been shown to be linked to metastatic activity of MDA-MB231 human breast cancer cells by increasing Ras/MEK/ERK and PI3K/Akt/mTORC1 signalling. The aim of this study was to investigate the potential of polymeric nanoparticles to deliver two novel shRNA sequences, targeted against the *RAN* gene, to MDA-MB231 cells grown in culture and to assess their effects in a range of biological assays.

**Methods:**

Biodegradable PLGA nanoparticles, loaded with shRNA-1 and shRNA-4, were fabricated using a double emulsion solvent evaporation technique and characterised for size, zeta potential and polydispersity index before testing on the MDA-MB231 cell line in a range of assays including cell viability, migration, invasion and gene knock down.

**Results:**

shRNA-loaded nanoparticles were successfully fabricated and delivered to MDA-MB231 cells in culture, where they effectively released their payload, causing a decrease in both cell invasion and cell migration by knocking down *RAN* gene expression.

**Conclusion:**

Results indicate the anti-*RAN* shRNA-loaded nanoparticles deliver and release biological payload to MDA-MB231 cells in culture. This works paves the way for further investigations into the possible use of anti-*RAN* shRNA-loaded NP formulations for the treatment of breast cancer in vivo.

## Introduction

Genetic instability in cellular function has a significant bearing on the transition from normal to cancerous growth patterns [1]. Of concern is the overexpression of mutated genes that enables tumour cells to metastasise to distant sites and establish secondary growth [2]. It is widely accepted that metastatic disease is associated with high rates of mortality observed in cancer patients and inhibiting metastasis is, therefore, subject to intense investigation [3]. These investigations focus on the function and mutation in specific genes [3]. Of particular interest to our group is the overexpression of the *RAN* gene in breast cancer cells, which has attracted much attention, with results demonstrating its clear role in the metastatic process [4]. This functional loss of cell cycle machinery causes cells to acquire aggressive and invasive characteristics, enabling invasion at distant sites.

Ran is a small GTPase involved in various cellular processes, including nucleocytoplasmic transport, apoptosis, mitotic spindle organisation and nuclear envelope formation [5,6]. Moreover, it is overexpressed in many human tumours, including stomach, lungs, head and neck, pancreas, ovarian, colorectal and kidney, but not in non-tumour tissue [4,7–14]. Importantly, RNAi-mediated knockdown of Ran in various tumour cell types cause aberrant mitotic spindle formation, mitochondrial dysfunction and apoptosis, while knockdown in non-tumour cells is well tolerated and does not lead to mitotic defects or loss of cell viability [9]. Therefore, overexpression of the *RAN* gene mediates invasiveness in human cancer cells, brought about by increasing Ras/MEK/ERK and PI3K/Akt/mTORC1 signalling [4,15]. Targeting the function of RAN is a feasible therapeutic target, leading to possible disruption in both cell proliferation and invasion [4]. This overexpression can be inhibited by RNA interference (RNAi), whereby the over-expressed gene is silenced using small oligonucleotide sequences, such as shRNA. However, the effective delivery of naked shRNA is problematic as it crosses the plasma membrane barrier poorly [16]. To circumvent this difficulty, cellular translocation of ribonucleic acid is assisted using nano-sized drug carriers, ranging from liposomes to polymeric nanoparticles (NP), which have been widely exploited in this field of therapeutics [17–21]. More specifically, polymeric NP, loaded with shRNA, have been shown to facilitate intracellular delivery [21]. Furthermore, the ability of polymeric NP to encapsulate and deliver a drug substance to the required target site without affecting its biological efficacy is a key advantage [22].

Recent studies show that viral-mediated shRNA or siRNA delivery inhibit cell invasion and metastasis by silencing *RAN* in MDA-MB231 human breast cancer cells [4,23,24]. For instance, lentiviral vectors have been used to deliver oligonucleotides that target *RAN* effectively [4] . Studies performed by the El-Tanani group demonstrate that this lentiviral mediated delivery of specific shRNA sequences, developed in their laboratory and known as shRNA-1 and shRNA-4, effectively silenced the *RAN* gene and inhibited MDA-MB231 cell invasion [4]. In addition to this, in silico analysis was carried out where shRNA-1 and shRNA-4 sequences complimented with the exons region on the targeted *RAN* gene, which provided further evidence of gene silencing via shRNA-1 and shRNA-4 mediated RNA interference. However, viral-mediated delivery is associated with toxicity and so an aim of this study was to use a simple polymeric vector based on poly(lactide-co-glycolide) (PLGA) [25]. This polymer has an acceptable toxicity profile and regulatory approval [26]. Results from recent studies show that NP prepared from PLGA and its PEGylated co-block variants (PLGA-PEG) are effective drug delivery vehicles for shRNA/siRNA and bring about silencing of targeted genes [27,28]. For example, the Bcl-xl gene in breast cancer cells was silenced using shRNA-PLGA-PEI NP and PLGA-siRNA NP have been effective in silencing a model gene (fire-fly luciferase) in MDA-kb2 cells [29–32]. Therefore, in this study, we report for the first time the encapsulation of two specific shRNA sequences (shRNA-1 and shRNA-4) in PLGA NP and demonstrate silencing of *RAN*. A modified double emulsion solvent evaporation technique was used to fabricate nucleotide-loaded NP with efficient encapsulation and these were evaluated using cell culture. The effects of both shRNA-loaded NP variants on MDA-MB231 human breast cancer cells were assessed by a range of in vitro assays, such as cell viability, migration assay, invasion assay and qRT-PCR, to determine the effect of *RAN* knockdown on metastatic potential.

## Materials and Methods

### Materials

Poly(d,l-lactide-co-glycolide) (PLGA) with a lactic:glycolic ratio of 50:50 (Resomer® RG 503H, MW 34 kDa), poly[(d,l-lactide-co-glycolide)-co-PEG] diblock (Resomer® RGP d 5055 (5% PEG) and Resomer® RGP d 50,105 (10% PEG)) were purchased from Boehringer-Ingelheim (Ingelheim, Germany). Poly(vinyl alcohol) (PVA) 87–89% hydrolysed (MW 31,000-50,000) and potassium chloride were purchased from Sigma-Aldrich (Dorset, UK).

shRNA-1 sequence clone IDs NM_006325.2-697s1c1 (sequence_CCGGGCACA GTATGAGCACGACTTACTCGAGTAAGTCGTGCTCATACTGTGCTTTTTG), shRNA-4 clone IDs NM_006325.2-697s1c1 (sequence_ CCGGGACCCTAACTTGGAATTTGTTCTC GAGAACAAATTCCAAGTTAGGGTCTTTTT) were purchased from Addgene (Cambridge, Massachusetts, USA). Quant-iT™ RiboGreen® RNA Assay Kit, RNA zap and diethyl pyrocarbonate (≥99%) (DEPC) were obtained from Thermo Fisher Scientific Ltd. (Paisley, UK). All the other chemicals and reagents were of appropriate analytical grade.

Cell culture studies were performed on the MDA-MB231 breast cancer cell line, which was obtained from Sigma-Aldrich Ltd. (Dorset, UK). Dulbecco’s Modified Eagle Medium (DMEM), foetal bovine serum (FBS), penicillin streptomycin, optimum reduced serum media, serum-free media and trypsin were obtained from Gibco, Life Technologies (Paisley, UK). Phosphate-buffered saline (PBS) was prepared from tablet form and purchased from Oxoid Ltd. (Hampshire, UK).

### Fabrication of shRNA-Loaded NP

NP loaded with shRNA (either shRNA-1 or shRNA-4) were fabricated using a double emulsion solvent evaporation technique, where a water-in-oil-in-water (w/o/w) emulsion was prepared. PLGA and diblock copolymers of PLGA and PEG containing 5% or 10% *w*/w of PEG 5 kDa were used in this work. Briefly, polymer (100 mg) was dissolved in 4.0 ml dichloromethane (DCM) and vortexed until fully dissolved. Thereafter, 1.0 ml of 2.50% (*w*/*v*) and 50 ml of 1.25% (w/v) PVA solution in DEPC-treated water (0.01% *v*/v) were prepared as internal aqueous phase and external aqueous phase, respectively. The payload (14 μg), of either shRNA-1 or shRNA-4, was dissolved in 1.0 ml of 2.50% PVA solution. This payload solution was added slowly and mixed with the polymer-DCM phase under constant homogenisation for 2 min to form the primary w/o emulsion. The primary emulsion was then mixed slowly with 50 ml of 1.25% of PVA solution to form a secondary w/o/w emulsion, which was kept under constant homogenisation (homogeniser-Silverson L5T FAR A17122) at 10000 rpm for 6 min at room temperature. Samples were left uncovered and stirred overnight at room temperature to allow evaporation of organic solvent. This was followed by a detailed three-part washing procedure, comprising centrifugation with (i) RNAse-free, DEPC-treated water, followed by (ii) 2% *w*/*v* sucrose solution and finally, (iii) with RNAase-free water. Repeated washing steps were carried out to remove any residual PVA and any shRNA that may have been absorbed onto the NP surface. After the final centrifugation step, the supernatant was discarded and the pellet dispersed in 5.0 ml RNAase-free ultra-pure water. Samples were freeze dried (Labconco freeze dryer, Mason Technology, Missouri, USA) before storage and use.

### Characterisation of shRNA-Loaded NP

Size, zeta potential and polydispersity index (PDI) of loaded NP were obtained using dynamic light scattering (Zetamaster, Malvern Instruments, Malvern, UK) employing a 15 mW laser and an incident beam of 676 nm. NP (5 mg) were dispersed in distilled water, filtered (0.45 μm) and used to determine mean diameter and size distribution. The measurement of zeta potential was performed on samples treated in a similar fashion, with NP dispersed instead in 1.0 mM potassium chloride solution.

The morphology of NP was determined using scanning electron microscopy (FEI Quanta 400 FEG, Eindhoven). An ultra-thin layer of lyophilised NP powder was coated on a carbon tape metal grid. It was then sputter coated with gold for 15 min and samples were examined with high vacuum mode and a 2° electron ETD detector.

### Determination of Drug Encapsulation Efficiency

The encapsulation efficiency (EE) of shRNA-loaded NP was determined using fluorescent nucleic acid staining (Quant-iT™ RiboGreen® RNA Assay kit, Thermo Fisher Scientific, Waltham, USA). Briefly, a sample of shRNA-loaded NP (5 mg) was dissolved in 1.0 M NaOH (0.25 ml) for 24 h under mild shaking and the pH adjusted to 7.0 using 0.5 M HCl. Dissolved samples were diluted in 1.0 ml TE buffer (10 mM Tris-HCl, 1 mM EDTA, pH 7.5) and then added to 1.0 ml of working reagent (1 part of ribogreen reagent in 20-fold of nuclease free DEPC-treated water). Samples were incubated in dark conditions for 15 min at 37°C and 200 μl from each transferred to a 96-well black plate and the fluorescence recorded (λ_ex_ 480 nm, λ_em_ 520 nm) using a microplate reader (FLUOstar Omega, BMG LabTech, UK). The analytical method was calibrated using a standard plot (0–1 μg ml^−1^ shRNA), performed as per the manufacturer’s instructions. The standard calibration curve was used to determine the amount of shRNA loaded per unit mass of NP (μg per mg of NP). From this data, the encapsulation efficiency was determined, defined as the percentage ratio of the determined nucleotide loading to the theoretical maximum loading.

### Drug Release Study

The drug release profile from loaded NP was determined by measuring the concentration of free shRNA in an aqueous receiver phase over a 96-h duration. To cover this 96-h period, four separate and identical samples were produced, each comprising shRNA-loaded NP (5 mg) suspended in PBS (1.0 ml, pH 7.4). All samples were rotated through an end-over-end motion, whilst being incubated at 37°C. At each 24 h-time point, a vial was selected, centrifuged at 10000 x g for 10 min, a sample of supernatant collected (200 μl) and the shRNA concentration measured using fluorescent nucleic acid staining, as described in section 2.4.

### Determination of Cellular Uptake of shRNA-Loaded NP

Cellular uptake of shRNA-loaded NP was determined by fluorescently labelling the nano-particulate core. Coumarin 6 (0.5 mg) was encapsulated within the NP by addition to the dichloromethane-polymer phase used during the double emulsion procedure. MDA MB 231 breast cancer cells (1 × 10^5^ cells per well in 1 ml media) were seeded in 2-well chamber slides and incubated for 24 h. Cells were then transfected by addition of 1 μg of shRNA-loaded NP dispersed in 1 ml transfection media (Opti-MEM® media) and then incubated for 24 h at 37°C. After 24 h, the media was aspirated and cells washed three times with PBS. Cells were then washed with 4% formaldehyde, stained with 100 μl of 0.5 mg ml^−1^ DAPI solution and visualised using fluorescence microscopy (Nicole Eclipse E400 Fluorescence Microscope, Nikon Y-FL, Japan).

### Cell Viability Assay

A suspension of 4 × 10^4^ cells in 1.0 ml medium (Opti-MEM®) was seeded per well in 24-well cell culture plates and left to adhere overnight at 37°C under a 5% CO_2_ atmosphere. After 24 h, the medium was aspirated and cells transfected with 500 μl of a nanoparticulate suspension in Opti-MEM® medium, at a concentration equivalent to 0.5 μg ml^−1^ (C1), 1.0 μg ml^−1^ (C2) and 2.0 μg ml^−1^ (C3) shRNA. Both shRNA-1 and shRNA-4 suspensions were prepared in this manner. Cell viability was evaluated after 24, 48, 72 and 96 h with the culture media being changed daily. Media were aspirated and cells washed with sterile PBS before addition of 500 μl media containing 15% (*w*/*v*) of an MTT dye solution (10 mg ml^−1^) in PBS solution was added to each well. All plates were incubated for 3 h at 37°C. The supernatant was then aspirated and formazan dissolved using dimethyl sulfoxide (DMSO) (500 μl per well). Absorbance was measured at 570 nm using a microplate reader (FLUOstar Omega, Ortenberg, Germany). Cell viability was calculated as a percentage of cell growth with respect to untreated cells and cells treated with blank nanoparticles.

### In Vitro Cell Migration Assay

A suspension of 7.5 × 10^5^ cells in 2 ml medium (Opti-MEM®) were seeded in 6-well plates and incubated at 37°C for 48 h. Once the cells had reached confluence, an induced artificial gap, or scratch, was made across the confluent cell monolayer using a sterile pipette tip. Medium was aspirated and cells transfected using nanoparticulate suspensions equivalent to 0.5 μg ml^−1^, 1.0 μg ml^−1^ or 2.0 μg ml^−1^ of shRNA dispersed in 2 ml Opti-MEM® media. Each well was viewed using light microscopy after 0, 24, 48 and 72 h to determine scratch closure, which was calculated using the formula below. Data were analysed using ImageJ.software (Cambridge, Massachusetts, USA).

$$ \%\mathrm{scratch}\ \mathrm{closure}=\frac{\mathrm{mean}\ \mathrm{width}\ \mathrm{at}\ \mathrm{time}\ 0-\mathrm{mean}\ \mathrm{width}\ \mathrm{at}\ \mathrm{time}\ t}{\mathrm{mean}\ \mathrm{width}\ \mathrm{at}\ \mathrm{time}\ 0}.100 $$

### In Vitro Cell Invasion Assay

A cell invasion assay was performed using a Cultrex® 96 Well BME Cell Invasion Assay kit. The assay was carried out according to the manufacturer’s instructions. Briefly, 50 μl 1x BME solution (basement membrane extract solution supplied with the kit) was added to the upper chamber of each well to produce a basement membrane-like structure and was incubated overnight at 37°C. After overnight incubation, the remaining solution was aspirated and wells were seeded at a density of 5 × 10^4^ cells per well in 150 μl serum-free medium into the upper chamber. Cells were incubated for 24 h at 37°C and 5% CO_2_ to allow adhesion to the basement membrane. Cells were then transfected with 2.0 μg ml^−1^ shRNA-loaded PLGA NP in 200 μl serum free media. Simultaneously, 150 μl of complete medium containing 10% foetal bovine serum was added to the bottom chamber to act as a chemo-attractant. Cells were incubated for 24 and 48 h at 37°C, 5% CO_2_, after which time the top chamber and bottom chamber were aspirated and wells were washed with 100 μl and 200 μl PBS washing buffer, respectively. Next, a solution of calcein-AM and cell dissociation solution were prepared by adding 12 μl of calcein-AM solution to 10 ml of cell dissociation solution, supplied with the kit, to detach the cells. Calcein AM/cell dissociation solution (100 μl) was added to the bottom chamber wells and incubated for 30 min at 37°C. The cell dissociation solution was used to detach invading cells from the membrane and calcein AM was internalised by viable cells. Esterase activity inside cells converted hydrophobic calcein-acetomethyl ester into hydrophilic fluorescent calcein. Later, the fluorescence of the bottom chamber was measured at 520 nm (485 nm excitation) using a microplate reader.

### qRT PCR-Gene Knockdown

A suspension of 1 × 10^5^ cells in 2 ml medium was seeded into each well of 6-well plates and incubated overnight at 37°C to ensure adherence. Cells were transfected using 1 μg of shRNA-loaded NP (shRNA-1 and shRNA-4) in 2 ml Opti-MEM® media and then incubated at 37°C for 24 and 48 h. After each time period, the media were aspirated and cells washed several times with PBS. Cells were trypsinised using 1.0 ml of 0.5% trypsin-EDTA 10X solution in each well and incubated at 37°C for 5 min. Growth medium (1.0 ml) was added into each well, then removed and the cell suspension centrifuged at 1500 x *g* for 5 min. The supernatant was discarded and the pellet resuspended with sterile PBS and centrifuged again at 1500 x *g* for 5 min. This was repeated twice. RNA was then isolated from MDA-MB231 cells using selective binding to silica-based membranes (RNeasy® Mini Kit, Qiagen Ltd., Manchester, UK). cDNA was synthesised from 2 μg of extracted RNA using forward and reverse primer sequences based on the mRNA sequence of the human *RAN* gene (forward primer 5’ CCATCTTTCCAGCCTCAGTC 3′; reverse primer 5’ TACCACCATCACCAACCAAT 3′).

### Statistical Analysis

All statistical analyses were performed using GraphPad PRISM software (GraphPad Prism, San Diego, USA). All experiments were performed in triplicate, unless otherwise stated. Data are presented as mean with standard deviation (SD) and significant differences were determined using a 2-tailed Student’s t test and calculated to one of three levels of significance, when appropriate.

## Results

### Physicochemical Characterisation of shRNA-Loaded NP

The key parameters of shRNA-loaded (shRNA-1 and shRNA-4) PEGylated and non-PEGylated NP are shown in Table I and define size, zeta potential, PDI and encapsulation efficiency. PLGA-shRNA NP were larger than the corresponding PEGylated equivalents for both the shRNA-1 and shRNA-4 formulations. Addition of PEG to the polymeric structure also affected the zeta potential and PDI of the NP formulations, with an elevation of zeta potential toward neutrality and a tightening of the distribution in mean diameter. However, the presence of PEG in the particle matrix did not significantly affect encapsulation efficiency, which was observed to exceed 66% for all formulations.

**Table I:** Physical characterisation of shRNA-loaded NP, where average size, zeta potential, polydispersity index (PDI), encapsulation efficiency (EE) and encapsulated drug amount were determined*

**Copolymers**	**size** **(nm)**	**PDI**	**zeta potential** **(mV)**	**EE** **(%)**	**Drug loading** **(μg per mg NP)**
PLGA-shRNA-1	426.41 ± 26.23	0.414 ± 0.144	−0.332 ± 0.66	74.94 ± 1.32	0.10 ± 0.01
PLGA-PEG-5%-shRNA-1	251.61 ± 14.71	0.231 ± 0.015	−0.224 ± 1.03	75.10 ± 7.67	0.10 ± 0.10
PLGA-PEG-10%-shRNA-1	272.36 ± 9.82	0.336 ± 0.018	−0.092 ± 0.37	66.31 ± 3.74	0.09 ± 0.05
PLGA-shRNA-4	408.45 ± 20.18	0.348 ± 0.012	−2.71 ± 0.039	85.18 ± 2.52	0.11 ± 0.03
PLGA-PEG-5%-shRNA-4	289.93 ± 13.24	0.201 ± 0.020	−1.81 ± 0.023	84.02 ± 5.81	0.11 ± 0.08
PLGA-PEG-10%-shRNA-4	237.71 ± 23.36	0.265 ± 0.011	−1.17 ± 0.044	80.01 ± 1.06	0.11 ± 0.02

*Data are mean ± SD with n = 6

**Fig. 1:**
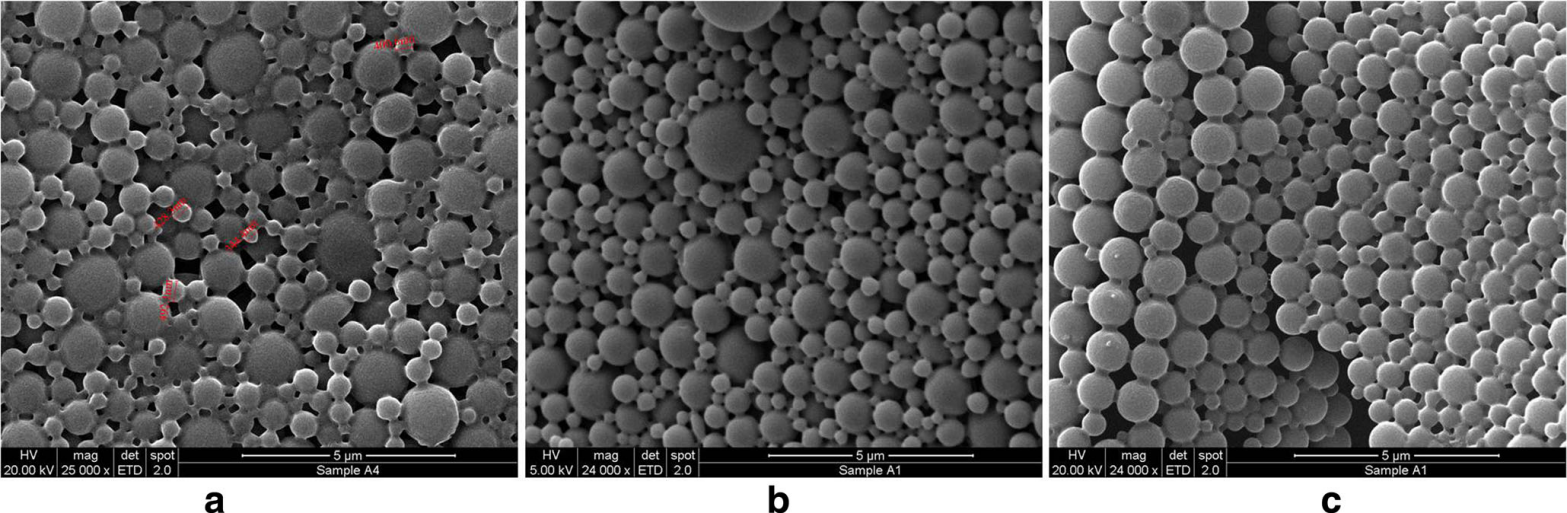
Surface morphology of loaded NP using scanning electron microscopy where (a) represents PLGA-shRNA-1 NP, (b) PLGA-PEG-5%-shRNA-1 NP and (c) PLGA-PEG-10%-shRNA-1 NP

The morphology of shRNA-loaded NP was determined using SEM and in Fig. 1 it is observed that both PLGA and PEG-PLGA NP display a smooth, spherical structure. An assessment of the mean diameters from these images is in close agreement with the dynamic light scattering data.

### Release Studies

**Fig. 2:**
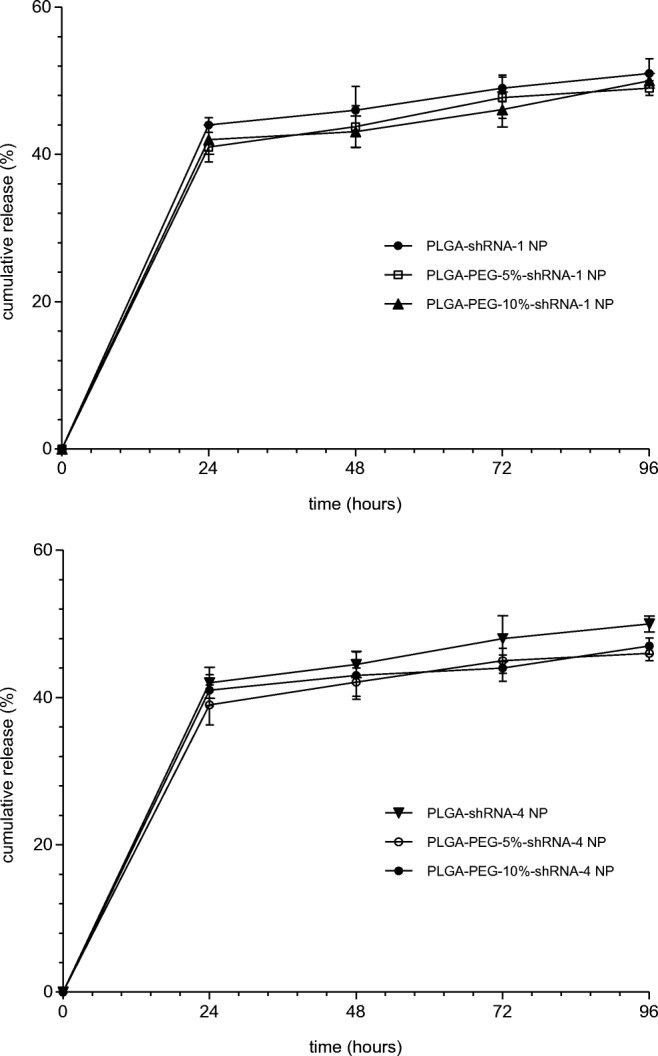
Cumulative release of shRNA-1 and shRNA-4 from loaded NP determined over a period of 96 h. (data are mean ± SD, with *n* = 3)

The division of the release profile into time-related phases, as often observed in nanoparticulate formulations for cancer applications [33], was evident in Fig. 2. Although complicated profiles are known, comprising possibly triphasic patterns, the data in Fig. 2 were observed to be biphasic, which is a common finding in PLGA NP systems [26,34]. The cumulative release of shRNA NP (shRNA-1 and shRNA-4 both) comprised the initial burst, when approximately 40% of the nucleotide loading was released in the first 24 h. This was then followed by a slower and more sustained phase, which is approximately zero order until 96 h. Differences in release between the two shRNA types was not apparent in the data, with both achieving a total release of approximately 51% after 96 h. Furthermore, the additional of PEG to the NP matrix did not produce any significant increase in the drug release over the observed experimental period.

### Cellular Uptake

**Fig. 3:**
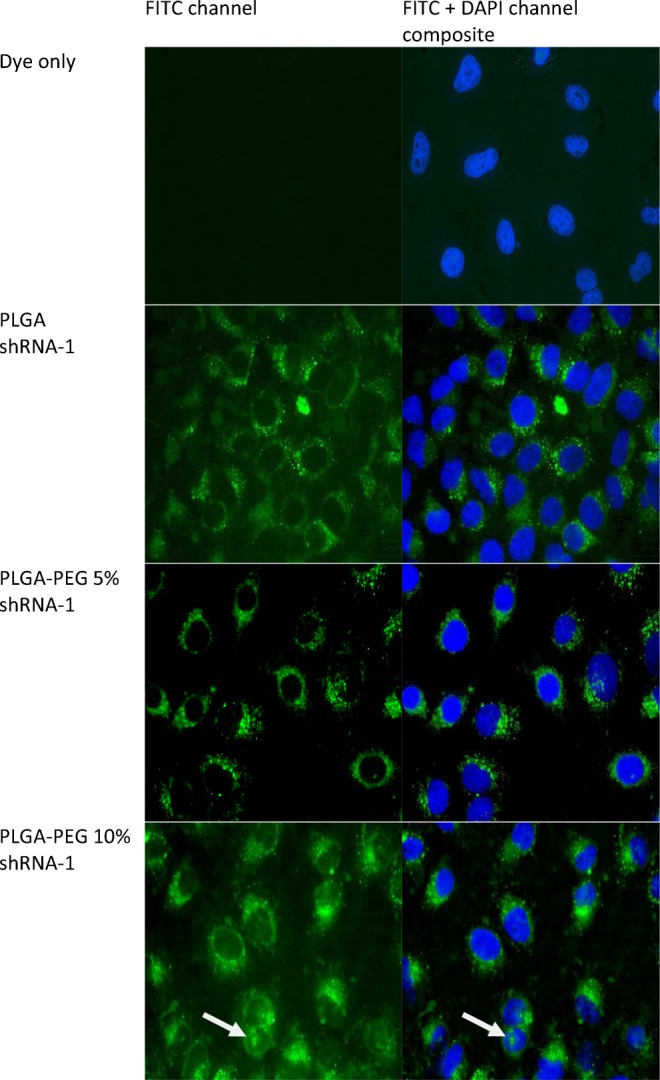
Intracellular localisation of coumarin-loaded shRNA-1 NP in MDA-MB231 cells. The DAPI channel (λ_ex_ 358 nm, λ_em_ 461 nm) was used to visualise staining of the cell nucleus and the FITC channel (λ_ex_ 488 nm, λ_em_ 519 nm) was used to visualise coumarin-6-loaded NP. Images taken from both channels were merged to determine the location of intracellular nanoparticulate localisation

Fluorescence imaging of coumarin-6-labelled NP following exposure to MDA-MB231 cells in shown in Fig. 3. Control experiments with free dye confirm effective nuclear staining, together with no observable cytoplasmic staining. Upon exposure to the fluorescent NP and upon inspection of the FITC channel, there is evidence of uptake into the cytoplasm. The nuclear space is dark, suggesting that nuclear uptake has not occurred. This observation would also support a lack of non-specific surface binding to the cell, which would be expected to light up the nuclear space, from either above or below the cell.

Merged images confirm no localisation of NP in the nuclear space. There is sporadic evidence of NP occurring within the nucleus (highlighted by the white arrow), but it cannot be confirmed if these NP are either bound to the outermost cell surface or have translocated across the nuclear membrane.

### Cell Viability

The results of the cell viability assay following exposure to shRNA-loaded NP are shown in Table II. Free shRNA and blank NP formulations did not cause any significant reduction (*P* < 0.05) in cell viability after 24 h. After longer periods, there was evidence of a modest reduction in viability, with the biggest drop observed (88%) after 96 h following exposure with PLGA-blank NP. Generally, no control formulation caused a drop in viability below 90%.

**Table II:** Viability of MDA-MB231 breast cancer cells following exposure to shRNA-loaded NP (shRNA-1 and shRNA-4)

	shRNA-1*				shRNA-4*			
	24 Hours	48 Hours	72 Hours	96 Hours	24 Hours	48 Hours	72 Hours	96 Hours
Cells	100 ± 3.1	100 ± 10.3	100 ± 6.5	100 ± 8.0	100 ± 3.1	100 ± 0.0	100 ± 7.0	100 ± 6.0
shRNA	101.6 ± 9.1	105.3 ± 8.2	99.7 ± 7.2	98.2 ± 8.2	99.6 ± 4.0	101.4 ± 3.0	100.7 ± 7.8	98.2 ± 8.2
PLGA-Blank NP	96.1 ± 11.1	92.1 ± 6.0	92.7 ± 6.0	88.1 ± 8.1	96.1 ± 0.8	92.1 ± 3.1	92.7 ± 4.1	88.1 ± 8.1
PLGA-PEG-5%-Blank NP	97.5 ± 10.0	92.0 ± 5.9	93.3 ± 6.1	94.1 ± 5.1	95.2 ± 2.9	94.6 ± 2.9	92.0 ± 3.8	93.1 ± 7.1
PLGA-PEG-10%-Blank NP	96.1 ± 10.0	95.0 ± 4.9	93.6 ± 6.0	90.1 ± 8.0	96.7 ± 4.4	95.1 ± 3.0	94.7 ± 4.3	90.0 ± 4.2
PLGA-shRNA-NP C1	85.6 ± 3.3	32.9 ± 11.7	63.9 ± 5.5	68.9 ± 7.3	80.6 ± 1.6	68.9 ± 6.4	74.7 ± 2.0	68.9 ± 7.3
PLGA-shRNA-NP C2	45.8 ± 10.-0	19.23 ± 6.5	34.2 ± 8.7	37.7 ± 16.8	82.0 ± 3.3	60.2 ± 4.8	71.1 ± 1.7	37.7 ± 16.8
PLGA-shRNA-NP C3	36.5 ± 5.5	16.0 ± 4.3	10.4 ± 4.2	8.0 ± 2.8	18.1 ± 0.5	41.2 ± 12.7	29.7 ± 3.4	10.0 ± 1.1
PLGA-PEG-5%-shRNA-NP C1	43.3 ± 5.8	32.2 ± 4.3	58.6 ± 10.0	45.3 ± 9.3	92.2 ± 5.0	92.5 ± 1.2	95.1 ± 8.8	78.9 ± 2.7
PLGA-PEG-5%-shRNA-NP C2	45.8 ± 7.1	31.5 ± 5.7	53.9 ± 1.2	42.9 ± 12.3	92.8 ± 4.6	79.2 ± 9.2	57.0 ± 4.4	47.7 ± 2.9
PLGA-PEG-5%-shRNA-NP C3	53.5 ± 4.8	38.3 ± 8.4	35.8 ± 3.4	33.4 ± 3.2	72.0 ± 1.26	82.6 ± 9.4	71.6 ± 1.8	18.0 ± 5.8
PLGA-PEG-10%-shRNA-NP C1	48.3 ± 7.1	32.2 ± 3.5	58.6 ± 9.9	45.3 ± 9.3	66.9 ± 8.6	80.5 ± 9.4	56.3 ± 10.5	50.7 ± 19.4
PLGA-PEG-10%-shRNA-NP C2	45.8 ± 5.0	31.5 ± 8.2	53.9 ± 0.7	42.9 ± 12.3	34.9 ± 7.2	74.9 ± 7.7	50.7 ± 6.4	30.1 ± 7.2
PLGA-PEG-10%-shRNA-NP C3	41.3 ± 6.0	30.9 ± 7.2	40.4 ± 6.9	41.2 ± 2.7	43.2 ± 3.3	58.4 ± 6.3	54.0 ± 4.9	25.4 ± 3.3

*Data are mean ± SD with *n* = 6

C1, C2 and C3 represents concentrations of shRNA used to transfect the cells, where C1 = 0.5 μg ml^−1^, C2 = 1.0 μg ml^−1^ and C3 = 2.0 μg ml^−1^ of entrapped shRNA

The encapsulation of shRNA into NP had a significant influence in cell viability. For example, cell viability reduced following transfection with increased amounts of shRNA-1 in PLGA NP. The highest concentration (C3) reduced viability to 36% after 24 h. When the addition of PEG to the NP matrix is considered, a similar reduction in cell viability was observed. This is seen in the data in Table II when PLGA-PEG-5% was used to deliver shRNA-1 to the cells. A significant decrease in cell viability was observed after incubation of the cells with PLGA-PEG-10% NP (***P < 0.001) using the lowest concentration (C1) of shRNA after 24 h, as more than 50% of the cell population was reduced. Interestingly, no further decreases in viability was observed following longer exposures. Without PEG, the effect of both longer exposure and higher concentration of shRNA-1 becomes more effective, with a cell viability of 8% using C3 and after 96 h.

Cell viability observed for shRNA-4 was generally in agreement to that determined for shRNA-1. However, there is greater influence of concentration and time of exposure for all formulations, with the presence of PEG having a greater effect. As with the shRNA-1 formulation, the PLGA NP with no PEG was found to have the biggest effect on viability (10%) after 96 h and at 2.0 μg ml^−1^ of entrapped shRNA-4.

### Cell Migration

**Fig. 4:**
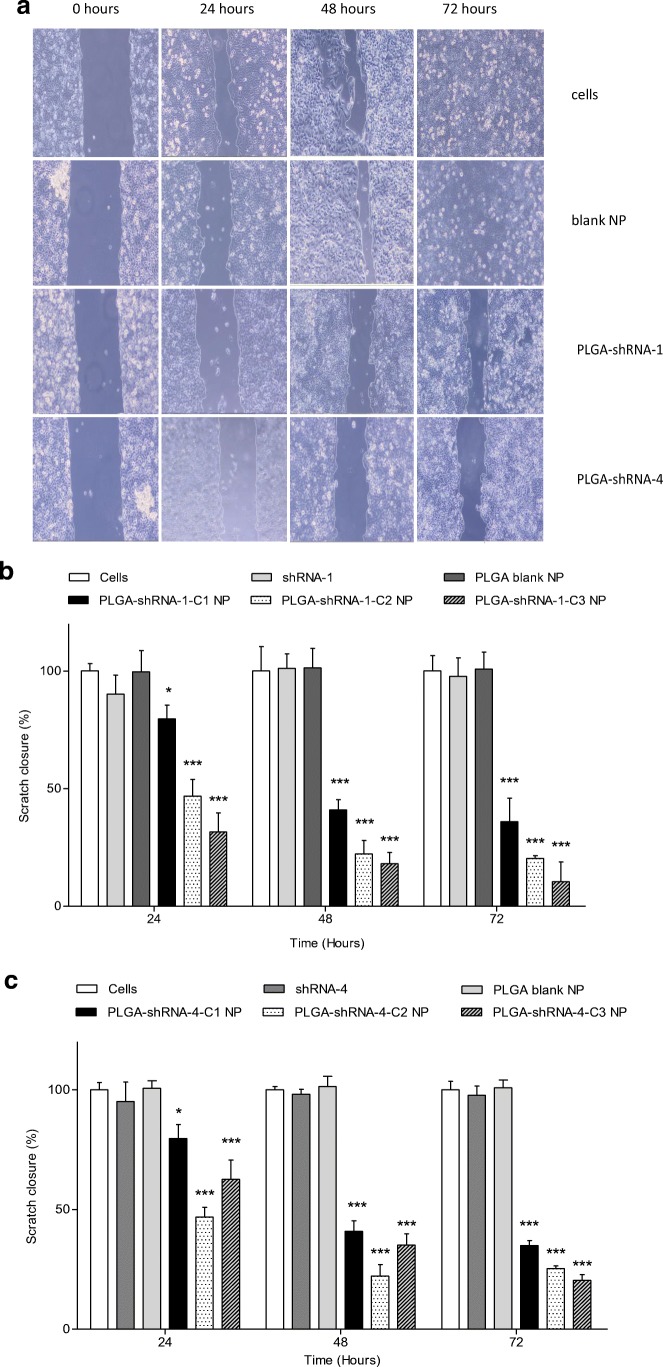
Scratch assay performed on confluent MDA-MB231 cells showing (A) light microscopy examination of the effect of PLGA-shRNA-1-NP and PLGA-shRNA-4-NP at 2.0 μg ml^−1^ on cell migration and (B) graphical representation of the dimensional change over 72 h using PLGA-shRNA-1-NP at three different concentrations and (C) graphical representation of the dimensional change over 72 h using PLGA-shRNA-4-NP at three different concentrations). Data are mean ± SD with *n* = 6. *P < 0.05, **P < 0.01, ***P < 0.001, C1, C2 and C3 are 0.5 μg ml^−1^, 1.0 μg ml^−1^ and 2.0 μg ml^−1^ of entrapped shRNA-4

The results from the cell migration assay confirm that scratch closure was significantly inhibited by both shRNA-1 and shRNA-4-loaded NP and the rate of migration was affected by the concentration of shRNA inside the NP. Free, non-encapsulated shRNA and blank PLGA NP did not have any effect on migration. Both Figs. 4(B) and 4 (B) confirm a concentration dependence on the % scratch closure, so that after 24 h, for example, 79% of the scratch width was observed in PLGA-shRNA-1 C1 treated cells, whereas with PLGA-shRNA-1 C3 treatment, the same response was found to be 34%. This result showed that cell migration was slower when more shRNA was available in the NP formulation. The data in Fig. 4 show that the patterns of cell retardation over time were similar for both shRNA types, with the shRNA-1 demonstrating the larger reduction in scratch closure after 72 h.

### Cell Invasion Assay

**Fig. 5:**
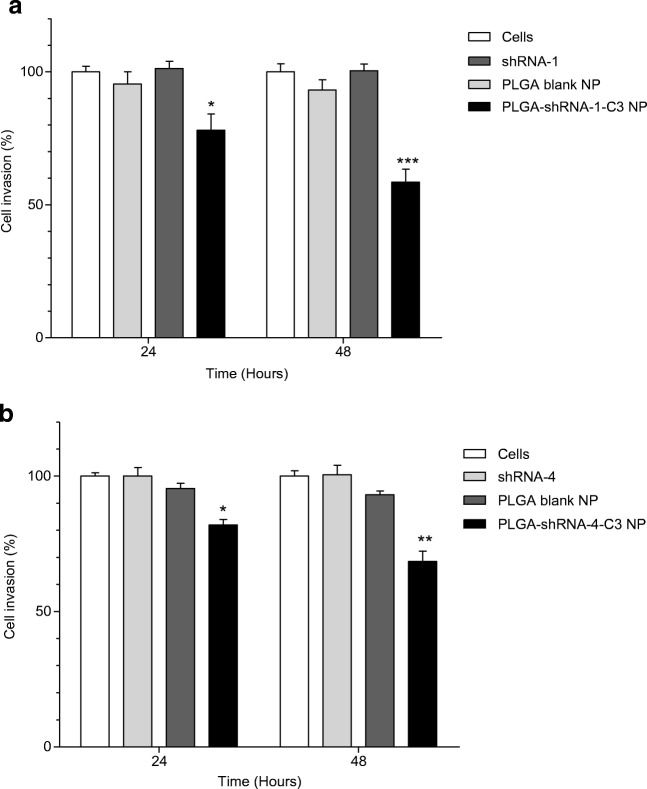
Effect of 2.0 μg ml^−1^ shRNA delivered using (A) PLGA-shRNA-1 NP and (B) PLGA-shRNA-4 on the invasion of MDA-MB231 cells. Data are mean ± SD with *n* = 3. *P < 0.05, **P < 0.01, ***P < 0.001, where significance is determined by comparison with invasion following treatment with blank NP

Invasiveness is a property of MDA-MB231 cells that contributions to their aggressive growth pattern. Attenuation of this characteristic is of particular interest in devising novel therapeutic interventions [35]. Therefore, an invasion assay was carried out to study the anti-invasive properties of shRNA-loaded NP and the results are shown in Fig. 5 . It was observed that PLGA-shRNA-1 NP (2.0 μg ml^−1^) inhibited significantly the invasion of the cells up to 57% after 48 h (***P < 0.001). In contrast, treatment with blank PLGA NP and non-encapsulated shRNA-1 showed no significant effect on invasion. Similarly, shRNA-4-loaded NP slowed the invasion of cells with a reduction in invasion of 20% after 24 h and 38% after 48 h, when compared to controls. A greatest significant effect is observed after 48 h and using shRNA-1 (P < 0.001). Although a reduction in invasiveness is significant after 48 h using encapsulated shRNA-4, the data in Fig. 5 (A) shows that is reduction is not as great as the result obtained using shRNA-1. Nevertheless, both shRNA types, when encapsulated in PLGA NP, reduced cell invasion.

### qRT-PCR-Gene Knockdown

**Fig. 6:**
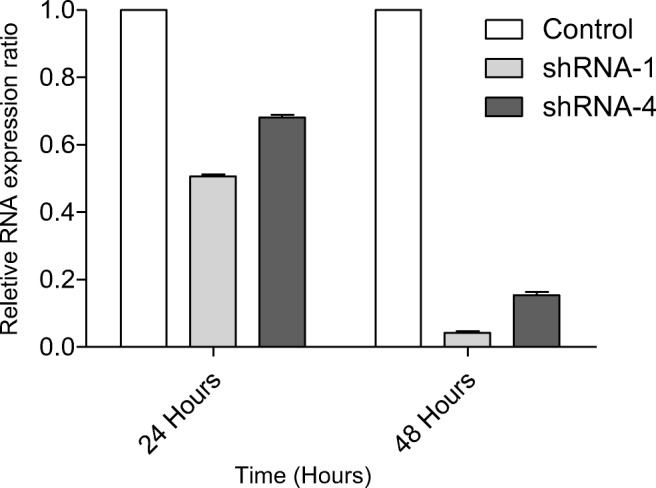
qRT-PCR for *RAN* gene expression after 24 and 48 h transfection of PLGA-shRNA-1 NP and PLGA-shRNA-4, where cells were transfected with 2.0 μg ml^−1^ shRNA-1 and 2.0 μg ml^−1^ shRNA-4. Control was blank PLGA-shRNA NP. Relative gene expression was measured using β-actin as a house keeping gene

In this work, real time quantitative PCR was used to determine the effect of shRNA-1 and shRNA-4 on *RAN* inside the cell. Knockdown (95%) of *RAN* was observed when cells were transfected with PLGA-shRNA-1 NP and knockdown of 85% was observed following transfection with PLGA-shRNA-4 NP after 48 h, as shown in Fig. 6 . In order to demonstrate a maximal effect, the concentration of shRNA (shRNA-1 and shRNA-4) used for transecting cells was 2.0 μg ml^−1^. The expression of *RAN* was reduced significantly in both cases, but encapsulated shRNA-1 was found to be more effective than shRNA-4 as evident from the percentage of reduction of *RAN* expression. These results demonstrate RNA interference [36] and verifies the specificity of the shRNA variants used in this work towards *RAN*.

## Discussion

Silencing of the *RAN* has been shown by our group to have significant potential in reducing both invasion and proliferation in an aggressive tumour cell line [4]. Effective delivery of shRNA, which is needed to bring about silencing, is a challenging task. However, polymeric nanoparticles have been shown to assist intracellular delivery of biological payloads, but key parameters, such as the polymer type, loading and release rates, must be investigated beforehand. This was especially pertinent in this work given that it is the first report of polymeric encapsulation of shRNA-1 and shRNA-4. Therefore, as part of the initial formulation investigations, PLGA and PLGA-PEG co-block polymers were chosen as the NP matrix. The use of PLGA is an understandable choice as it is widely used for drug delivery applications, being both biodegradable and non-toxic. However, in order to optimise drug loading and subsequent release, alternations in the polymer packing and hydrophobicity are required. A common approach to do this is to use co-block addition of PEG residues, which was the approach adopted in this work. This provides a means to adjust particle size, charge and loading and is often part of the formulation studies used to optimise encapsulation efficiency and cellular uptake.

The key parameters investigated in this work, such as size, charge and drug loading are shown in Table I. Mean diameter plays a significant role in cellular uptake, with NP in the range of 50–500 nm taken up more efficiently than larger NP via endocytotic pathways [37,38]. However, given the difficulties in the delivery of naked shRNA to cells, the key focus of this work was the loading of NP with shRNA and the effective cellular delivery of the payload, not the size of the NP themselves. The double emulsion procedure used in the work was able to produce NP with a mean size within this range. Differences in the sizes of shRNA-loaded PLGA and PLGA-PEG NP were due to the difference in the chain length of PLGA polymeric moiety in the polymer [39]. On adding PEG to the PLGA polymer, the hydrophobic chain size of PLGA was condensed, which reduced the hydrophobic aspects of the NP core and the size the NP. This effect has been reported for other smaller molecular weight drug substances, such as flurbiprofen-loaded PLGA NP (190 nm), which were bigger than flurbiprofen-loaded PLGA-PEG NP (170 nm) [39].

The surface charge on a NP plays an important role in its interaction with the plasma membrane of cells. Since the plasma membrane is anionic, it will favour the absorption of cationic NP due to electrostatic interaction [40,41]. PLGA-shRNA, PLGA-PEG-5%-shRNA and PLGA-PEG-10%-shRNA NP (both shRNA-1 and shRNA-4) all possessed a negative surface charge, PLGA-shRNA exhibited the maximum negative charge followed by PLGA-PEG-5%- shRNA and PLGA-Peg-10%-shRNA. Addition of PEG to PLGA masked the negative charge on PLGA-PEG-shRNA nanoparticles and hence makes them less negative. This masking effect increased on increasing the PEG concentration in the PLGA-PEG polymer moiety. The results in Table I show that inclusion of PEG to the PLGA NP did reduce the negative zeta potential but this caused an increase in cell viability rather than a decrease as was expected (Table II). This finding has been observed in other work [42]. The data in Table I also shows that the double emulsion procedure is able to achieve impressive encapsulation efficiencies in nanoparticles that are spherical and smooth (Fig. 1). Similar studies have reported encapsulation efficiencies of siRNA in PLGA NP of approximately 51%, comparing favourably with the findings of this current study, which demonstrated efficiencies of around 80% for the PLGA NP [43,44]. However, it was observed that the encapsulation of the payload increased when PEG increased, suggesting that enhanced hydrophilicity did not improve encapsulation. Clearly, the presence of PEG in the NP core does not lead to enhanced incorporation.

The release of encapsulated drugs from NP can be sustained, or adopt a burst release, or both, depending on the NP formulation type [45]. The profiles in Fig. 2 show similarity between all formulations tested. There are indications that PLGA-shRNA-NP give higher release than PLGA-PEG-shRNA NP, but this finding is not significant. Other work has shown better release from PLGA NP when compared to PEG-PLGA NP and it has been proposed that this effect arises due to hydrophilic interaction between the drug and PEG moieties, leading to a delay in the diffusion of the drug [46]. However, Fig. 2 shows a distinctive burst release phase during the first 24 h, which is attributable to release of surface resident and adsorbed shRNA. The slower phase, occurring after 24 h and for several hours afterwards, is attributable to NP degradation and diffusion through the NP core [46].

Intracellular uptake of drug-loaded NP is an appealing property, especially if subsequent release occurs directly into the cytoplasm. Our group has described the development of two novel shRNA sequences, as used in this work, which if delivered intracellularly, can exert anti-proliferative and anti-invasive effects in MDA-MB231 breast cancer cells. Non-encapsulated forms of our shRNA sequences (shRNA-1 and shRNA-4) without assistance from delivery vectors are ineffective and did not affect cell viability. Similar results were obtained elsewhere when non-encapsulated shRNA was shown to be ineffective at transfecting LNCaP prostate cancer cells, whereas retroviral-mediated shRNA delivery caused inhibition in cell populations [47]. In this current work, blank NP were found to be non-toxic, whereas shRNA-loaded PLGA NP, shRNA-loaded PLGA-PEG-5% NP and shRNA-loaded PLGA-PEG-10% NP significantly reduced cell viability, as shown in Table II. Cell viability decreased with an increase in concentration of NP (C1 < C2 < C3). Cell viability increased at the 72 h point for PLGA-shRNA-NP C1 and some other of the formulations, which may be due to the temporal release profile of the nanoparticles, as suggested by Basu et al. [48]. They reported a similar increase in cell viability when MAPK inhibitor-loaded PLGA nanoparticles were delivered to MDA-MB231 cells. Another study by Wang et al. [49] also reported an increase in cell viability after the fifth day of a cell viability study after introduction of nanoparticles and they suggested this increase was due to the high encapsulation efficiency of the nanoparticles.

The results from fluorescence imaging, as shown in Fig. 3, suggests that these NP have translocated to the cytoplasm. PLGA was shown to be superior to its PEG-containing variants, with shRNA-1 and shRNA-4 encapsulated in PLGA NP significantly reduced cell viability to 8% and 10% after 96 h, respectively (***P < 0.001) when the highest drug loading was used (C3 = 2.0 μg ml^−1^). This reduction in viability was concentration dependent, particularly in the PLGA formulations. This form of concentration dependency is a common finding. For example, bicalcutamide-loaded PLGA NP in various concentrations (0.5 μg ml^−1^ – 50 μg ml^−1^) to C4–2 prostate cancer cells caused a proportional reduction in cell viability in relation to the payload concentration [50,51].

An important aim of this work was to demonstrate that shRNA-1 and shRNA-4 inhibited the migration of MDA-MB231 breast cancer cells following nanoparticulate delivery. In order to rationalise the experimental design, the formulations providing the most effective reduction in cell viability (PLGA-shRNA-1 and PLGA-shRNA-4) were taken forward for evaluation of migration and invasion. The scratch assay was used to quantify migration using a simple measure of determining gap closure, as shown in Figs. 4 and 5. Results in these figures show that both PLGA-shRNA-1 and PLGA-shRNA-4 brought about a concentration-dependent reduction in the speed at which MDA-MB231 breast cancer cells migrate and close the induced gap. shRNA-1-loaded NP slowed migration significantly when compared to blank NP, non-encapsulated shRNA-1 and untreated cells (controls). Indeed, 72 h was sufficient time to allow the scratch to close completely when no treatment was given. However, the gap was still apparent following treatment with loaded NP. Results for cell invasion followed a similar pattern to migration. Invasion of MDA-MB231 cells was significantly reduced to 60% (*P* < 0.001) and 68% (*P* < 0.01) after 48 h following treatment with PLGA-shRNA-1 NP and shRNA-4 NP, respectively, with shRNA-1 marginally superior to shRNA-4.

The data in Fig. 6 confirm that *RAN* knockdown was observed when cells were transfected with PLGA-shRNA-1 NP and PLGA-shRNA-4 NP with relative expression rations of 96% and 85%, respectively. Effective knockdown of *RAN* gene demonstrates that shRNA is undergoing endosomal escape after internalisation of the NP which is crucial it these delivery vehicles are to have a therapeutic potential. These figures compare favourably to reported values of 90% knockdown of *RAN* using viral vector in pancreatic and melanoma cell lines [52,53]. Importantly, the concentration of shRNA used in these studies was 6 μg ml^−1^, which was 3 times higher than the concentrations used in this study. This comparison demonstrates the effective delivery using the PLGA carrier and is comparable to the levels achieved with more established viral vectors.

## Conclusion

PLGA NP loaded with two novel shRNA sequences were of a size range that has been shown to be effective in cell uptake. The encapsulation efficiency was found to be high for all formulations with minimal loss of the payload during the nanoparticle fabrication. Variation in the PEG content allowed for adjustment in particulate properties, such surface charge and size distribution. However, the inclusion of PEG did not improve in vitro characterisations, such as migration and invasion, with pure PLGA NP loaded with both shRNA-1 and shRNA-4 producing significant reductions in migration and invasion of MDA-MB231 breast cancer cells. However, differences in shRNA-1 and shRNA-4 in terms of drug release, cell viability and *RAN* knockdown were small, with the former being judged as marginally superior. The results of this study demonstrate that shRNA-1 and shRNA-4 can be delivered effectively using PLGA NP and that biological activity is preserved once drug release has occurred. This delivery system is a potential therapeutic means to cell invasion and migration in cells of metastatic potential.
